# Artificial Trabecular Meshwork Structure Combining Melt Electrowriting and Solution Electrospinning

**DOI:** 10.3390/polym16152162

**Published:** 2024-07-30

**Authors:** Maria Bikuna-Izagirre, Javier Aldazabal, Javier Moreno-Montañes, Elena De-Juan-Pardo, Elena Carnero, Jacobo Paredes

**Affiliations:** 1 Tissue Engineering Group, Tecnun School of Engineering, University of Navarra, Manuel Lardizabal 13, 20018 San Sebastian, Spain; mbikunai@unav.es (M.B.-I.); jaldazabal@unav.es (J.A.); 2 Biomedical Engineering Center, University of Navarra, Campus Universitario, 31080 Pamplona, Spain; 3 T3mPLATE Harry Perkins Institute of Medical Research, QII Medical Center, 6 Verdun St., Nedlands, WA 6009, Australia; elena.juanpardo@uwa.edu.au; 4 UWA Center of Medical Research, The University of Western Australia, 35 Stirling Highway, Perth, WA 6009, Australia; 5 Navarra Institute of Health Research, IdisNA, Calle Irunlarrea 3, 31088 Pamplona, Spain; ecarnero@unav.es; 6 Ophthalmology Department, University of Navarra Clinic, Avenida PIO XII, 31080 Pamplona, Spain; jmoreno@unav.es

**Keywords:** melt electrowriting, human trabecular meshwork, solution electrospinning, glaucoma, tissue engineering

## Abstract

The human trabecular meshwork (HTM) is responsible for regulating intraocular pressure (IOP) by means of gradient porosity. Changes in its physical properties, like increases in stiffness or alterations in the extracellular matrix (ECM), are associated with increases in the IOP, which is the primary cause of glaucoma. The complexity of its structure limits the engineered models to one-layered and simple approaches, which do not accurately replicate the biological and physiological cues related to glaucoma. Here, a combination of melt electrowriting (MEW) and solution electrospinning (SE) is explored as a biofabrication technique used to produce a gradient porous scaffold that mimics the multi-layered structure of the native HTM. Polycaprolactone (PCL) constructs with a height of 20–710 µm and fiber diameters of 0.7–37.5 µm were fabricated. After mechanical characterization, primary human trabecular meshwork cells (HTMCs) were seeded over the scaffolds within the subsequent 14–21 days. In order to validate the system’s responsiveness, cells were treated with dexamethasone (Dex) and the rho inhibitor Netarsudil (Net). Scanning electron microscopy and immunochemistry staining were performed to evaluate the expected morphological changes caused by the drugs. Cells in the engineered membranes exhibited an HTMC-like morphology and a correct drug response. Although this work demonstrates the utility of combining MEW and SE in reconstructing complex morphological features like the HTM, new geometries and dimensions should be tested, and future works need to be directed towards perfusion studies.

## 1. Introduction

**Figure 1:**
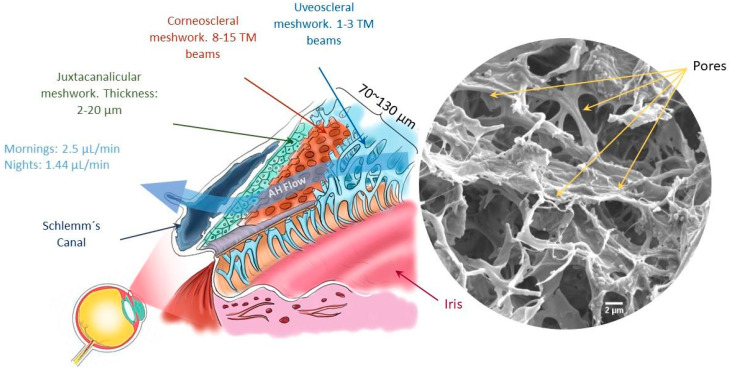
Location of the HTM. The anatomical structure of the tissue, which is formed by three layers: uveal, corneoscleral, and juxtacanalicular meshwork. Scanning electron microscopy image of the native trabecular meshwork tissue. Scale bar: 2 μm. Reprinted from M.Bikuna-Izagirre, J. Aldazabal et al. (2022) [6].

The human trabecular meshwork (HTM) is a tiny porous tissue responsible for intraocular pressure (IOP) regulation. IOP builds up mainly in response to resistance provided throughout the HTM to the aqueous humor (AH) outflow [1]. The HTM is made up of connective tissue containing collagenous and elastic fibers, and it is covered by flat cells [2]. This permeable tissue is located around the cornea and is composed of three different layers: the uveoscleral region (UVM), with pore sizes varying from 70 to 100 µm; the corneoscleral region (CTM), with pore sizes of 30 µm; and the juxtacanalicular region (JTC), with pore sizes of 4–7 µm (Figure 1) [3]. The pores are formed by cells and the extracellular matrix (ECM), organized in lamellar beams with a thickness of 5–12 µm [1]. The HTM, with a reported full thickness of 70–130 µm, and with CTM as the main compartment, also includes the UVM region, encompassing constructs of 40.6 ± 10.0 µm thickness [4], and the JCT region, with elements 2–20 µm thick [4]; it offers increasing resistance to fluid outflow across the varying thicknesses in the direction of the eye’s surface (from the inner to outer parts) as its porosity decreases [1]. Changes in the physical properties of the HTM, such as increases in stiffness (from 4 kPa in healthy tissue to 80 kPa in glaucomatous tissue) [4,5], alterations in ECM protein expression, or loss in cellular reparative capacity, can decrease the overall porosity of the HTM, resulting in AH outflow difficulties, a condition closely related to glaucoma [1,2].

Current research in the field is hampered by the lack of a more realistic in vitro HTM model, all present instances of which are based on one-layered models [6]. A whole three-layered architecture can allow repeatability in outflow studies and improve the current understanding as to how the structure and function are correlated in the natural tissue [6]; also, it can lead to regenerative solutions and therapies to assess diseases. In the literature, different scaffolds used as in vitro HTM models have been reported, addressing this need [6]. A microfabricated SU-8 membrane of pores 12 µm in size, although simple, was able to show the correct phenotype in terms of morphology, as well as the expression of the specific markers and ECM secretions of human trabecular meshwork cells (HTMCs) [7,8,9,10]. Despite successful attempts at replicating the in vivo outflow physiology, the HTMCs were only exposed to stiffer environments than found in the native tissue [11], and due to the overall construct thickness (<20 µm), they were not able to migrate into a 3D environment [12]. Other groups have worked towards 3D models based on hydrogels of collagen type I-elastin peptides, in which the pathological state was successfully induced by dexamethasone treatment and counteracted by rho-associated kinase (ROCK) inhibitor [12,13]. Collagen and collagen/chondroitin sulfate scaffolds obtained via freeze-drying were also used to build 3D HTM models. After 14 days of culturing, porcine trabecular meshwork cells were able to proliferate throughout the structure [14]. Morphological changes have been pursued, varying the pore size and alignment, and greater cell growth was found on larger pores, which indicates the key role the environment has in setting cell behavior [15,16]. In a similar manner, substratum stiffnesses have also been tested to study HTMC responses. As the stiffness of the substrate increases, so do the cellular focal adhesions, fibronectin and α-SMA expression levels, which are the two main HTMC-linked markers [11,17]. A 3D culture using Matrigel [17,18] showed promising results in overcoming chronic oxidative stress, with improved results in dynamic cell culture conditions. Despite the morphological and environmental modifications hydrogels offer, none of these studies take into account the layered structure of the HTM to recapitulate the in vivo tissue morphology [6]. Recently, a novel approach has been published, which includes a variation in pore size to improve cellular infiltration within the scaffold using cryo-electrospinning technology [14]. Despite the positive results, the scaffold did not present porosity variations throughout its depth, limiting the structure to a one-layered design. Another promising study includes a layered structure using melt electrowriting (MEW) technology [19]. A graded porous architecture was fabricated containing up to 88 stacked layers of polycaprolactone (PCL), where HTMCs successfully attach and proliferate for 14 days [19]. However, this approach is rather time-consuming, due to the required printing time. The number of layers and the square-like pores (different from the rounded morphology found in the native tissue) hinder the cellular infiltration towards inner layers and showed a higher elastic modulus than native HTM. Although standing as the closest approach to the complete native HTM, scaffolds that better reproduce native morphological features, allowing us to understand the structure–function relationship, are still needed. 

It is evident that there is a requirement for a multiphasic or porous-gradient scaffold for an in vitro engineered HTM. Two main electrospinning techniques have become popular among tissue engineering applications. On the one hand, solution electrospinning (SE), commonly known as electrospinning, is a well-known technique based on the electrostatic interactions between a polymeric solution and the extrusion needle to generate micro/nanofibers [20,21]. On the other hand, melt electrowriting (MEW), a marriage between SE and 3D printing, can generate microfibers with custom-designed patterns after melting the polymeric pellets [22]. There are numerous applications where electrospinning techniques have been used in the bioengineering field. For instance, SE has been used for vascular [23], bone [24], or tendon [25] applications. Similarly, MEW has been used to print thermoplastic polymers (PCL) into structures with different pore architectures, including squared [26], rectangular [27], dodecagon [28], or sinusoidal [29] architectures, for several applications such as skin [30], cardiac [27], and ligament [29] tissue engineering. However, the literature is scarce when it comes to HTM models using SE or MEW [31]. 

Focusing on the challenging graded-porous architecture of the HTM, the goal of this study was to present an innovative and novel method of combining MEW and SE for an HTM platform. To accomplish this, the topology, porosity, and mechanical properties of the scaffolds as a function of the design were characterized. For an in vivo-like physiology, HTMCs were seeded and their proliferation was studied. Moreover, the HTM model was treated with well-documented drugs: the glucocorticoid dexamethasone (Dex), known for its glaucomatous effects (increase in ECM proteins and MYOC overexpression) [32,33], and the rho inhibitor Netarsudil (Net) (AR-13324), which has shown positive results in reducing the IOP due to the loss of focal adhesions and the cytoskeleton disruption [34,35,36]. Thus, our aim was to determine whether the combination of SE and MEW can support HTMCs and enable their response to drugs as described in the literature. This study paves the way to combining two of the most promising technologies to develop functional biomimetic in vitro models, allowing for a better understanding of the structure–function relationship in native HTM. 

## 2. Materials and Methods

### 2.1. Fabrication of SE and MEW Scaffolds: Printing Parameters

Scaffolds were fabricated using an in-house-built SE [32] and MEW [37] device made from PCL 80,000 kDa (Sigma Aldrich, St. Louis, MO, USA) and medical-grade PCL (Purasorb PC 12, Corbion, The Netherlands), respectively. TM1 and TM2 refer to UVM and CTM, respectively. TM3 was built using SE and represents the JCT. TMFull is the assembly of TM1, TM2, and TM3. For this process, SE nanofibers were deposited on an aluminum foil. Then, the foil was placed on the printing bed of the MEW machine; printing over the nanofibers began with TM2 first and TM1 afterward.

For the SE process, PCL (10 wt. %) was dissolved in chloroform/methanol (Panreac, Barcelona, Spain) at a volume rate of 4:1, *v/v* by agitating the mixture overnight (600 rpm) at room temperature (21 ± 1 °C). The polymeric dissolution was extruded through a 20 G metallic needle at a 0.5 mL h^−1^ rate with a syringe pump (Chemyx Fusion 100, Stafford, TX, USA). A high-voltage DC power supply (FC Series 120-watt, CE Compliant) was used to provide the necessary electric field between the needle and the collector. A voltage of 13 kV was applied with a distance of 10 cm between the needle and the collector. The nanofibers were collected on a 9 × 9 cm^2^ flat aluminum foil. 

The specific processing parameters used for the MEW process were air-pressure-driven extrusion at 156 kPa; through a 23 G metallic needle; at working distances of 4 mm for TM1 and TM2 and 5 mm for TMFull; a translational speed of 300 mm min^−1^; with a voltage of 4.4 ± 1 kV applied to the spinneret and grounded collector plate; and with a 86 °C ring heater and 30.5 °C bed heater. The exact processing parameters that were used were varied slightly depending on the environmental conditions on the given day. 

### 2.2. Scaffold Imaging and Measurements

Scaffolds were cut and fixed to SEM stubs using double-sided carbon tape. Stubs were then sputter-coated with palladium for 75 s, using an Emitech Mini Sputter Coater Quorum SC7620 (Quorum, Laughton, East Sussex, UK). SEM images were then acquired using Zeiss Gemini (Oberkohen, Germany) at an acceleration voltage of 5 kV, with a high probe current and high vacuum mode. Diameter and porosity measurements were taken on the bases of SEM images by using image analysis software (Image J v 1.54, NIH, Maryland, USA) and a Diameter J plugin (Nathan Hotaling—v1.018). 

### 2.3. Uniaxial Tensile Testing

Scaffolds were mechanically tensile tested across the interface using the ZwickLine Z1.0 (Zwick/Roell, Ulm, Germany) equipped with a 50 N load cell. Samples (*n* = 4 for each scaffold type) were cut to a width of 10 mm and secured with clamps with a separation of 20 mm between them. Uniaxial tests used a displacement rate of 0.1 mm/seg. Stress–strain curves were plotted using the generated force–displacement data, where strain was defined as engineering strain (change in length/initial length). The initial length was kept constant at 20 mm for all calculations. The cross-sectional area was quantified, keeping the width of the scaffolds constant (10 mm) and measuring the thickness with a digital gauge (Digimatic Serie 547, Mitutoyo, Tokyo, Japan) (n = 4 in each scaffold). Averages for each layer were as follows: TM1 = 2.6 ± 1.5 mm^2^, TM2 = 6.1 ± 2.6 mm^2^, TM3 = 0.20 ± 0.01 mm^2^, and TMFull = 5.1 ± 2.0 mm^2^. Young´s modulus was calculated from the slope of the stress–strain curve at the steepest linear region. Yield strength was calculated as the point where the stress–strain curve plateaued.

### 2.4. G-Code Generator GUI

An in-house program was developed using Python 3.12.0 (Python Software Foundation, USA) to incorporate continuous interface design into the spatially heterogeneous G-code for MEW printing. A script generating a GUI was created, allowing the desired parameters of the scaffold to be entered. Once entered, the interface would execute other scripts that perform the necessary calculations to plot data for the desired printing path. These data were then converted by another script into a G-code Notepad file for input into the custom-made MEW printer software.

### 2.5. Cell Experiments

Human donor eyes were obtained from the Center for Medical Research and Eduction (Universidad de Navarra, Spain). In total, 23 HTM donors (12 women and 13 men) with an average age of 71 ± 15.1 were obtained (patient medical histories not available). Primary HTMCs were isolated as indicated in Du Y et al. [38] Cells from two donors were used for the experiments. Briefly, the HTMCs were initially seeded on a 0.1% gelatin-coated 75 cm^2^ cell culture flask with high-glucose DMEM with 10% fetal bovine serum (FBS). Fresh culture medium was supplied every 48 h. Cells were maintained at 37 °C in a humidified atmosphere with 5% carbon dioxide until 80–90% confluence, at which point they were trypsinized using 0.05% Trypsin EDTA (Gibco, ThermoFisher, Waltham, MA, USA) and subcultured. All studies were conducted using cells before the 4th passage. 

Scaffolds were clamped in 24-well-plate cell crowns (Scaffdex, Tampere, Finland) and used for the experiments. The samples were used in triplicate. All scaffolds were treated with a plasma (100 W for 1 min, with 5 and 15 sccm of O_2_ and Ar, respectively) process (Diener Electronic, Ebhausen, Germany) to increase the hydrophilicity of the surface and sterilized prior to cell seeding in UV radiation for 30 min on each side. Subsequently, the scaffolds were coated with gelatin 0.1% for 30 min, and HTMCs were seeded at 10 × 10^4^ cells per well. The seeded scaffolds were kept for 14 days and 21 days, with the cell culture medium being changed every 2–3 days. Scaffolds cultured for 14 days were used for live/dead assays on days 1, 8, and 14, whereas 21-day cultures were used for drug treatment experiments. 

#### 2.5.1. Cell Viability

A live/dead cell viability assay was performed following the manufacturer´s protocol (Invitrogen, Waltham, MA, USA). This entails using cell media solutions of 2 µL/mL ethidium homodimer-1, to stain dead cells in red (excitation/emission ~ 535/617 nm), and 2 µL/mL calcein AM, to stain live cells in green (excitation/emission ~490/515 nm). Samples in triplicate on days 1, 8, and 14 were removed from the medium, placed in a fresh well plate, and incubated with 500 µL of staining solution for 45 min. After DPBS washing, cells on the scaffold were imaged with a Leica fluorescence microscope. Three images per sample were used for analysis. The images were analyzed with ImageJ software. After brightness and contrast adjustment, cell counting was performed with “Analyze particles”. The cell viability was calculated using the following equation: % viability = (no. of live cells/total no. of cells) × 100. 

#### 2.5.2. Nuclei Shape and Scaffold Infiltration

On days 14 and 21, samples in triplicate were fixed with paraformaldehyde (PFA) 4% for 30 min, followed by three washes in DPBS. After permeabilization with 0.3% Triton-X 100 for 20 min and another DPBS wash, cells were stained with 1:500 DAPI in PBS and washed 3 times with DPBS. Imaging was performed with a Leica fluorescence microscope (Leica, Germany). Three independent images at 20 X magnification were used to analyze nuclei parameters: length and width, and the ratio of length to width, further denoted as AR (aspect ratio). The images were processed with ImageJ software by color threshold adjustment, followed by watershed processing and the use of the “analyze particles” function (size limit 50–150 µm) [19]. Scaffold infiltration by cells was investigated using a confocal microscope (Zeiss LSM 980) and imaging in the z-stack mode. 

#### 2.5.3. Cell Culture with Glucocorticoids and Rho Inhibitors

On day 14, samples in triplicate were treated with Dex (Thermo Fisher Scientific, Waltham, MA, USA) at 15 nM [18] and Net (AR-13324) (rho inhibitor) at 1 µM [36] (CymitQuimica S.L., Barcelona, Spain) for 5 days. The medium was changed every 2–3 days, and samples were collected for SEM and fluorescence studies. 

#### 2.5.4. Immunostaining and Confocal Imaging

Cells were fixed and stained for the F-actin cytoskeleton, focal adhesion, and nucleus. After days 14 and 21, the cells were fixed with PFA 4% for 30 min, followed by three washes in DPBS. After permeabilization with 0.3% Triton-X 100 for 20 min, the cells were blocked with 2% bovine serum albumin in PBS. HTMCs were incubated overnight with the primary antibody mouse-anti-Vinculin (Proteintach, Manchester, UK, 1:300 dilution). Next, the cells were treated with the secondary antibody goat-anti-mouse Cy3 (Jackson Inmuno Research, West Grove, PA, USA 1:500 dilution) and Phalloidin AlexaFluor 488 (1:200) (Abcam, Amsterdam, The Netherlands) for F-actin staining for 2 h at room temperature. Finally, DAPI (1:500) was used to stain cell nuclei. 

Laser scanning confocal microscopy was performed using a Zeiss LSM 980 confocal microscope (Zeiss, Oberkohen, Germany), and images were acquired at 10X and 25X magnifications with an oil-immersion objective. Confocal images were processed using Zen Blue 3.10 software, and all confocal images within a given experiment were captured using the same laser intensity and gain settings to enable comparisons of intensities across samples. 

#### 2.5.5. SEM of Cell-Loaded Scaffolds

Similar imaging conditions as described above were used for scaffolds without cells. Cell-loaded scaffolds were fixed with 4% PFA for 30 min at room temperature. The samples were rinsed three times in DPBS and dehydrated in a graded ethanol series (50%, 70%, 80%, 95%, and 100%) for 5 min each at room temperature, then air-dried overnight. Fixed samples were mounted on stubs and sputter-coated with palladium at 18 mA for 75 s. Images were obtained at an accelerating voltage of 5 kV.

### 2.6. Statistical Information

Data were expressed as the mean ± SD. Statistical differences were analyzed using one-way analysis of variance (ANOVA) performed by GraphPad Prism software v9.3 (Graph Pad Software, La Jolla, CA, USA) (* *p*-value < 0.05, ** *p*-value < 0.01, *** *p*-value < 0.001, and **** *p*-value < 0.0001). 

## 3. Results and Discussion

### 3.1. Fabrication and Mechanical Characterization of Graded Porous Scaffold

Inspired by the distinctive architecture of the HTM, characterized by its varying pore sizes and consecutive layers, four scaffolds were designed: TM1, TM2, TM3, and TMFull. Each name corresponds to the layers of the HTM from the outer to the inner part: TM1 refers to the UVT, TM2 to the CTM, TM3 to the JCT region, and TMFull is a combination of these three layers. The targeted morphology featured circular shapes, mimicking the typical trabecular pattern, with high porosity and effective pore sizes decreasing in the order TM3 < TM2 < TM1.

**Figure 2:**
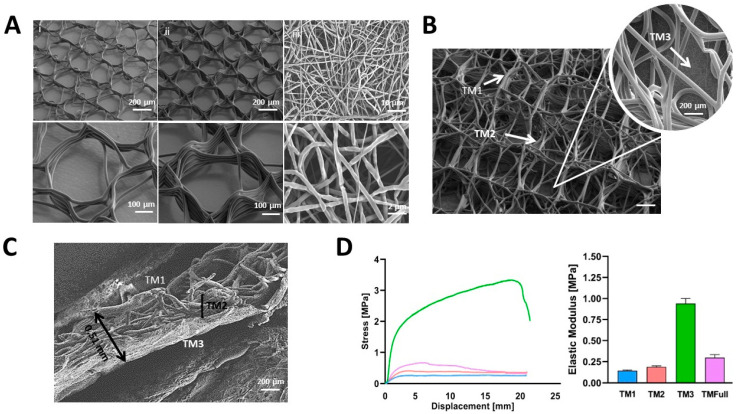
(**A**) PCL scaffolds representing (i) TM1 and (ii) TM2. Upper row scale bar: 200 µm, lower row scale bar: 100 µm. (iii) SE scaffolds representing TM3. Upper row scale bar: 10 µm, lower row scale bar: 2 µm. (**B**) SEM image of TMFull. Scale bar: 200 µm. (**C**) Transversal cut of TMFull scaffold. Scale bar: 200 µm. (**D**) Displacement–stress curves for each scaffold layer and linear elastic modulus (average over *n* = 4 samples for each scaffold design) Blue: TM1, Red: TM2, Green: TM3, Purple: TMFull.

Printing parameters were optimized to achieve the best shape and fidelity, despite circles being one of the most challenging shapes for MEW printing [39]. The scaffolds’ height, fiber diameter, pore size, and mechanical properties of each layer are summarized in Table 1. SEM images in Figure 2A–C confirm the morphological analysis. TM1 and TM2 exhibited well-defined circles (Figure 2A(i–ii)), with minimal defects, except for the last layers, where some fibers crossed the circles, reducing their diameter. In TMFull (Figure 2B and cross-section in Figure 2C), lower precision in fiber deposition was unavoidable. Varying circle diameters between levels and wall tilting led to alterations in the electrical field, causing control loss of the jet. Attempting to print a complex morphology with different height proportions resulted in more irregular material flow and bending of the deposited fibers, giving the scaffold a messy and chaotic appearance. The measured porosity was greater than the theoretical porosity for all the MEW scaffolds (Table 1) due to repulsive forces and difficulties in achieving the optimal critical translational speed. Consequently, larger pore areas than expected were achieved in TM1 and TM2, obtaining the desired porosity gradient. Further research in optimizing fabrication parameters for circular morphologies is needed. Alternatively, exploration of easier-to-control architectures via MEW might also yield interesting results. The mismatch of pores between TM1 and TM2 gave TMFull an interconnected appearance, with the outermost pores (TM1) crossed from above by TM2 fibers. Porosity in TM3 could not be controlled as it is a randomized process, but homogeneity and bead-free fibers were achieved.

The stress–strain curves from the uniaxial tensile test of the scaffolds are shown in Figure 2D. The curves display an initial linear region with a steep slope, followed by a plateau region. Except for the TM3 electrospun scaffolds, which reached their breaking point at 100% elongation, the remaining structures did not break below 200% elongation. The tensile modulus for all the designs ranged from 0.14 to 0.94 MPa (Table 1), consistent with previously observed values for MEW PCL-based scaffolds, which reach a range of a few MPa [19,40]. The moduli of TM1, TM2, and TMFull were similar, with the highest modulus found in the TM3 nanofibrous scaffold, likely due to its lower porosity and greater number of fibers. In MEW scaffolds (TM1 and TM2), failure occurred in consecutive single fibers, whereas in the nanofibrous scaffolds (TM3), breakage was more abrupt.

Different values have been reported for native HTM after dissection. Tensile tests of HTM segments showed a Young’s modulus of 515 ± 136 kPa, while porcine HTM showed 25 kPa [41]. Some studies showed values ranged from 4 kPa in normal tissues to 80 kPa in glaucomatous ones [5], and others values ranged from 3 to 52.6 MPa for glaucomatous HTM and 51.5 ± 13.6 MPa for normal eyes [42]. The literature shows varied results, which vary strongly depending on the measurement methods. The results obtained here show lower values than in previously reported MEW-based HTM (5.6–13 MPa) [19], which might not be problematic for physiological studies and could be addressed by reducing the pore size (changing the morphology) and increasing the fiber amount in each layer. A compromise between the scaffold´s thickness and cellular infiltration may be considered, as the former can hinder cell infiltration, nutrient diffusion, and waste removal. 

### 3.2. Cell Culture Studies

**Figure 3:**
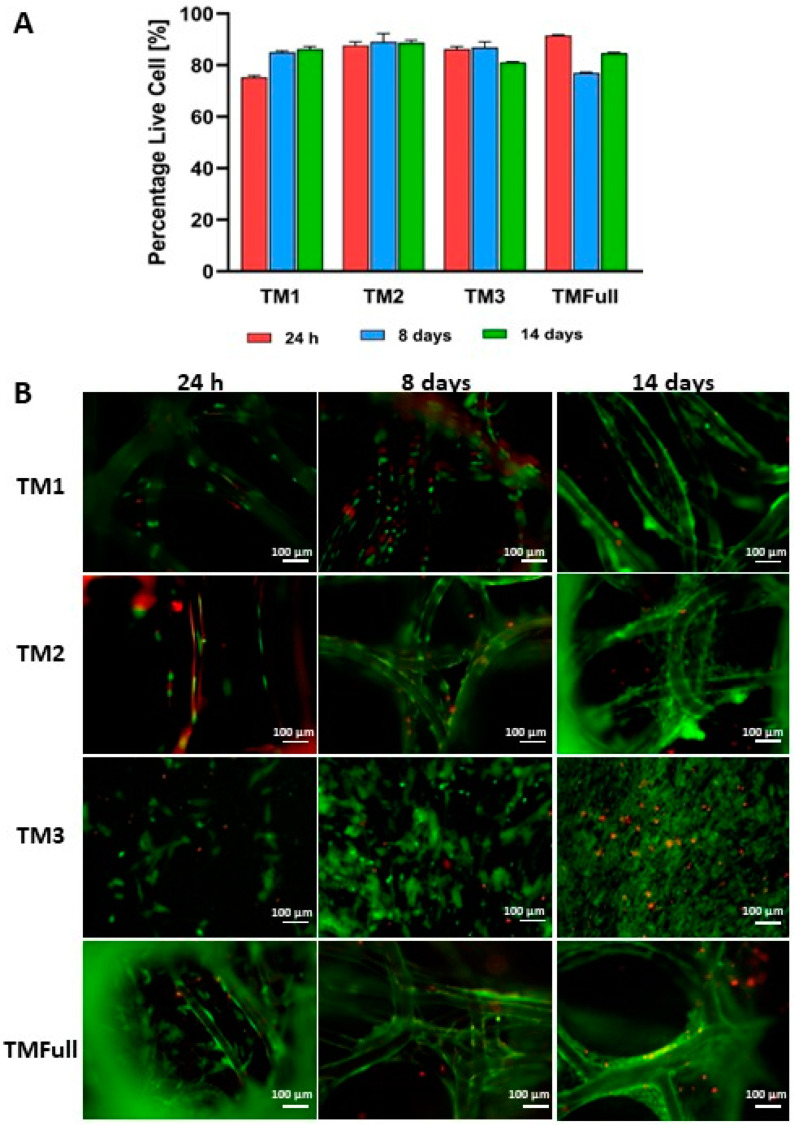
Biological evaluation of the cells growing on different scaffolds. (**A**) Bar plot showing the viability of the cells based on live/dead assay staining. (**B**) Fluorescence images of live/dead assay. Red color indicates dead cells and green the live ones. Scale bar: 100 µm.

Primary HTM cells were seeded on the scaffolds to test their ability to proliferate. We performed a live/dead assay to assess cell viability, followed by fluorescence imaging. In TM1, TM2, and TMFull samples, the stained cells were visible through the scaffold. However, for TMFull, which contained a higher number of layers, only cells in the more superficial layers were accessible for examination and included in the analysis. Cell viability ranged from 75% to 95% at days 1, 8, and 14 after seeding (Figure 3A). TM1 and TM2 showed similar trends, likely because larger pore sizes provide space for cells to spread and proliferate. A relatively lower viability was observed in TM3 and TMFull over time, possibly due to the lack of space in denser and more packed structures, which impedes cell media diffusion. This could lead to cell death in deeper layers (TMFull) and in the monolayer in the case of TM3 (due to lack of space) (Figure 3B). It is important to note that inaccuracies in the measurements might occur due to light reflection caused by the scaffolds.

#### 3.2.1. Cell Morphology and Distribution on the Scaffold

**Figure 4:**
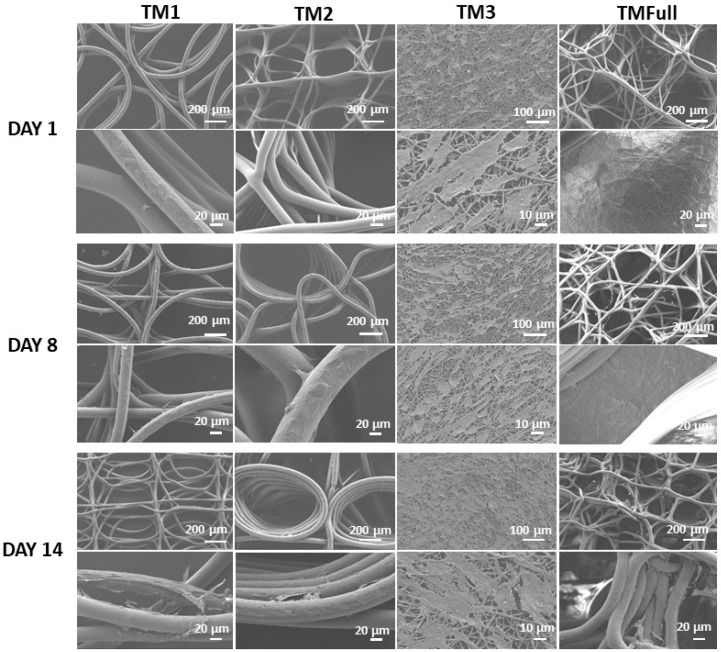
SEM images of scaffolds with HTM cells on day 1, day 8, and day 14 for morphological evaluation of the cells. Two magnifications were taken: top with scale bars: 200 µm and bottom scale bar: 20 µm. For TM3 samples, the scale bars were 100 µm and 10 µm, respectively.

The morphology of HTM cells on scaffolds at days 1, 8, and 14 after seeding was studied using SEM (Figure 4). On day 1 (Figure 4—Day 1), cells were observed accumulating at the nodes and on top of MEW fibers or where fibers crossed, with minimal spreading, especially evident in TM2. By day 8, cells had spread out on the scaffolds, exhibiting a typical spindle-like shape characteristic of native HTM cells. In TM3 (Figure 4—Day 8), cells formed a monolayer, whereas TM1 and TM2 showed less confluence, with proliferation primarily around the MEW fibers and their intersections. TM3 displayed a higher accumulation of cells throughout the layer, leading to fewer cells on the outermost fibers. The covered area was larger in TM3 compared to the other layers, with cells adopting elongated morphologies around the fibers. By day 14, TM1, TM2, and TMFull (comprising the MEW layers) exhibited increased cell presence around the MEW fibers, with some cells bridging between fibers (Figure 4—Day 14). In contrast, TM3 showed some empty areas, possibly due to limited space for cell proliferation. This observation is consistent with the findings from the live/dead assay (Figure 3A).

The SEM investigation (Figure 4) and tracking of nuclei distribution with confocal microscopy (Figure S1A) confirmed that cells successfully infiltrated all the scaffolds. After 14 days, F-actin staining revealed elongated structures on all scaffolds (Figure S1A), indicating that cells were attached to the scaffolds and able to develop cytoskeletal actin fibers. The most uniform infiltration was observed in the more densely packed scaffolds (TM3 and TMFull). Smaller pore sizes facilitated early bridging of fibers and the formation of confluent cell layers. Comparative studies using commercial membranes have shown that cells face challenges growing on larger pore sizes [43]. Previous research with SU-8 planar scaffolds indicated that cell proliferation is hindered in pore sizes larger than 15 µm [8]. In contrast to these 2D scaffolds [8,43] and hydrogel-based 3D models [13,14], where cell diffusion is limited, our model demonstrates improved cell infiltration due to pore sizes that allow for adequate media diffusion and provide a larger surface area for cell attachment. Hydrogel scaffolds with pore sizes around 200 µm have been found to enhance cell infiltration compared to those with pore sizes around 20 µm, likely due to the availability of alternative routes for cell proliferation and migration. Then, non-aligned pores were also more beneficial, which may lead to alternative routes for cells to proliferate and migrate [15]. In summary, our scaffold design with non-aligned pores and suitable pore sizes supports effective cell infiltration and growth, as evidenced by SEM, confocal microscopy, and F-actin staining results.

HTMCs in the native tissue have elongated cell shapes and elongated nuclei [8]. To further analyze HTMCs within the scaffolds, nuclei size in different scaffolds was evaluated based on DAPI staining images after 14 days of culturing. The aspect ratio (AR) parameter was calculated as a ratio of nucleus length to width. This method was described in [20], and in Figure S2, an example of the quantification is provided. The nuclei of the cells were elongated, as is typical in the native HTM (AR ≥ 1.5: 1.62 ± 0.3 for TM1, 1.67 ± 0.3 for TM2, 1.84 ± 0.3 for TM3, and 1.67 ± 0.6 for TMFull) (Figure S1C). Previous works reported values of 1.1 for porous membranes and 2.0 in an SU-8 photoresist membrane [8].

#### 3.2.2. Biological Characterization of PCL-Based HTM Scaffold

Morphological changes were observed in HTMCs after Dex (15 nM [32]) and Net (1 µM [34]) treatment over 7 days. The effect of Dex on HTMCs has been acutely studied [32,33], with reports of an increase in F-actin fibers, ECM material deposition, cell nuclei size growth, and IOP. Net instead regulates the cell contraction, cytoskeletal organization, adhesive interactions, and thus HTM permeability, ultimately regulating the IOP. At the cellular level, it induces cell shape changes, disruption of actin stress fibers due to focal adhesion loss, and alteration to the ECM composition [34,35,36].

**Figure 5:**
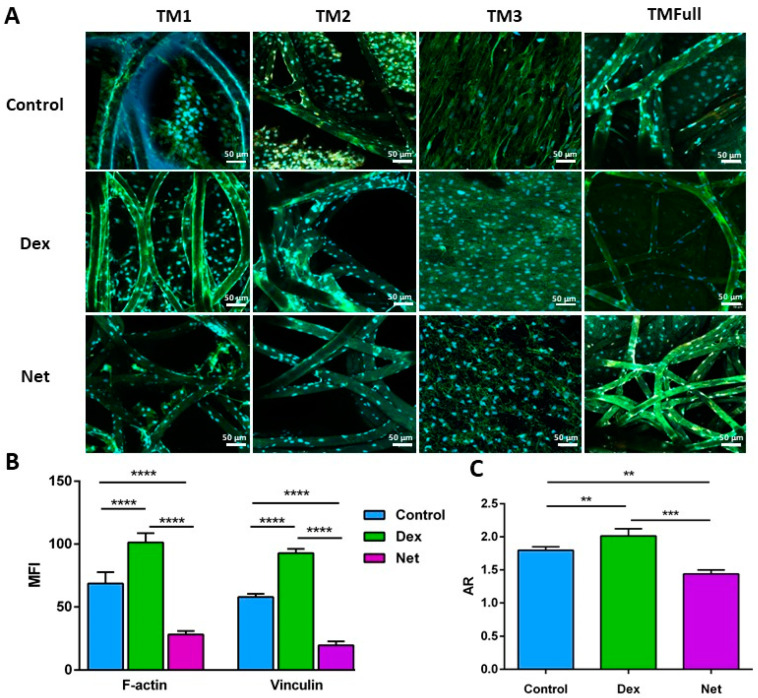
(**A**) Confocal images of HTM cells cultured for 21 days in different scaffolds—TM1, TM2, TM3, and TMFull—treated with Dex and Net. In green, F-actin fibers (AlexaFluor 488); orange, vinculin; and in blue, DAPI for nuclei staining. Scale bar: 50 µm. (**B**) Quantification of confocal images and mean fluorescence intensity (MFI), and (**C**) nucleus aspect ratio (* *p*-value < 0.05, ** *p*-value < 0.01, *** *p*-value < 0.001, and **** *p*-value < 0.0001).

F-actin fibers play a crucial role in regulating HTMC contractility and are a therapeutic target for lowering IOP. As expected, treatment with Net led to disassembly of the stress fibers, resulting in diffuse green patches of intracellular staining (Figure 5A). In contrast, treatment with Dex caused an increase in the mean fluorescence intensity (MFI) of F-actin fibers, as observed in both the confocal images (Figure 5A) and quantified data (Figure 5B). The shape of nuclei was assessed using DAPI after 21 days of culturing. In control samples, nuclei were elongated, with an aspect ratio (AR) of 1.82 ± 0.12, typical of native HTMCs [19] (Figure 5C). Dex treatment resulted in an increase in nucleus size (AR = 2.07 ± 0.07), likely due to glucocorticoid-induced alterations in these cells [44]. On the other hand, Net-treated samples showed a more rounded nucleus, with a reduced AR of 1.36 ± 0.22, reflecting the effects of rho inhibitor-based treatment [31,45] (AR values shown in Figure 5C). These observations highlight the ability of cells to alter their nucleus morphology in response to different treatments, demonstrating that the porous-graded PCL scaffold supports appropriate cellular behavior. It is important to note that the data presented herein represent averages from each layer (TM1, TM2, TM3, and TMFull) under each condition (control, Dex, and Net).

**Figure 6:**
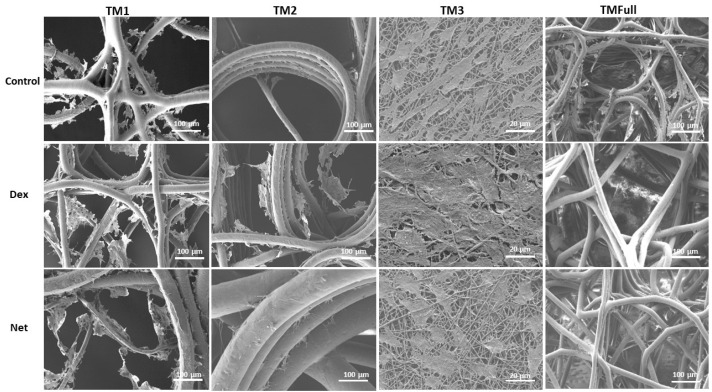
SEM images of HTM cells for morphological evaluation. Cells were cultured with cell media for 14 days and the subsequent 7 days with drugs. First row, control samples using cell media. Second row, 15 nM of Dex. Third, 1 µM of Net. Scale bars: 100 µm and 20 µm for TM3 case.

The changes in the cell morphology were also observed in the SEM images shown in Figure 6. Dex samples showed an increase in cell size (in each layer), where bridging between MEW fibers was more evident for TM1 and TM2. TM3 instead showed fewer empty spaces while almost covering the whole field of view (88.16 ± 9.35% of the image, indicating an increase in cell size). The reaction of HTMCs to Net showed a disrupted morphology due to the loss of focal adhesions, resulting in 21.57 ± 5.86% of the image being covered. In this case, less bridging between MEW fibers is observed in TM1 and TM2, and more free spaces between cells in TM3. This observation is consistent with previous in vitro studies indicating that our bioengineered HTM model is responsive to IOP-lowering drugs [19,31,44,45,46,47]. Finally, the control case in TM3 covered 40.49 ± 4.37% of the image. Statistical differences were found among the three groups (*p* < 0.05).

## 4. Conclusions

This study demonstrates that is possible to combine MEW and SE technologies to construct an in vitro HTM model for investigating HTMC behavior and conducting further outflow studies. Different PCL scaffolds were printed to recapitulate the different layers of the HTM. The combination of these three layers (TMFull) showed a graded-porous architecture, characteristic of the native tissue. The pore size of the constructs and the initial cell concentration influenced cell attachment, surface coverage, and the subsequent monolayer formation. Each layer, as well as the TMFull, remained stable for 14 and 21 days, supporting primary HTMCs, as demonstrated by cell morphology, nuclei size, and proliferation assays. The in vitro biological studies showed that the PCL scaffolds responded to Dex and Net treatments and confirm the central role of F-actin filaments and focal adhesions in maintaining the cell morphology. Future work can explore new architectural designs such as reducing the pore size in MEW scaffolds to create a more compact structure and enhance cell-monolayer formation. Additionally, to evaluate scaffold functionality, perfusion studies and the corresponding cellular analysis could be performed. To the best of our knowledge, this study presents the first biomimetic functional HTM model combining MEW and SE for the HTM and opens a door to conducting further research on the structural and cellular relationships in glaucoma and AH outflow. 

## Figures and Tables

**Table 1:** Scaffold height, fiber diameter, pore sizes (theoretical and the one measured after SEM analysis), elastic modulus, and yield stress.

			Pore Size			
	Scaffold Height [µm]	Fiber Diameter [µm]	Theoretical *	Measured	Elastic Modulus [MPa]	Yield Stress [MPa]
TM1	260 ± 15	37.5 ± 2.5	0.6 mm	0.86 ± 0.21 mm	0.14 ± 0.01	0.24 ± 0.04
TM2	610 ± 26	29.1 ± 1.7	0.4 mm	0.75 ± 0.15 mm	0.18 ± 0.01	0.39 ± 0.03
TM3	20 ± 1.4	770 ± 0.2 nm		5.59 ± 0.68 µm^2^	0.94 ± 0.05	2.84 ± 0.20
TMFull	510 ± 20				0.29 ± 0.03	0.65 ± 0.22

* Theoretical pore size makes reference to the size introduced in MEW’s software. TM3 does not have a theoretical measurement because SE is a randomized process and pore sizes cannot be predetermined.

## Data Availability

The original contributions presented in the study are included in the article, further inquiries can be directed to the corresponding author.

## Supplementary Materials

The following supporting information can be downloaded at https://www.mdpi.com/article/10.3390/polym16152162/s1, Figure S1: (A) Confocal images of HTM cells after 14 days of culturing on different scaffolds: TM1, TM2, TM3, and TMFull. The first channel represents the focal adhesions (vinculin first antibody and cy3 conjugate), the second channer represents phalloidin F-actin fiber staining (AlexaFluor 488), the third column represents DAPI for nuclei staining, and the last one represents the merge. Scale bars: 100 μm. (B) Quantification of confocal images and mean fluorescence intensity (MFI), and (C) nucleus aspect ratio. Figure S2: Exemplary image processing used for nuclei aspect ratio quantification. (A) Original nuclei image stained with DAPI. (B) Image made binary. (C) Image after applying “watershed” function. (D) Nuclei considered in the calculation after applying a size exclusion limit (50–150 μm). Note that the nuclei in the case of clamps of cells or overlapping were excluded from the calculation.
