# Supplementary material for: Artificial Trabecular Meshwork Structure Combining Melt Electrowriting and Solution Electrospinning
Source: Polymers (Basel). 2024 Jul 30;16(15):2162. doi: 10.3390/polym16152162 (PMC11314991; doi:10.3390/polym16152162)
Supplement: Supplementary file 1 [file polymers-16-02162-s001.zip › polymers-3092630-supplementary.pdf]

Supporting Information

Artificial Trabecular Meshwork Structure Combining Melt Electrowriting and Solution Electrospinning

Maria Bikuna-Izagirre [1,2,3,4], Javier Aldazabal [1,2,5], Javier Moreno-Montañes [6], Elena De-Juan-Pardo [3,4], Elena Carnero [5,6] and Jacobo Paredes [1,2,5*]

E-mail: jparedes@unav.es

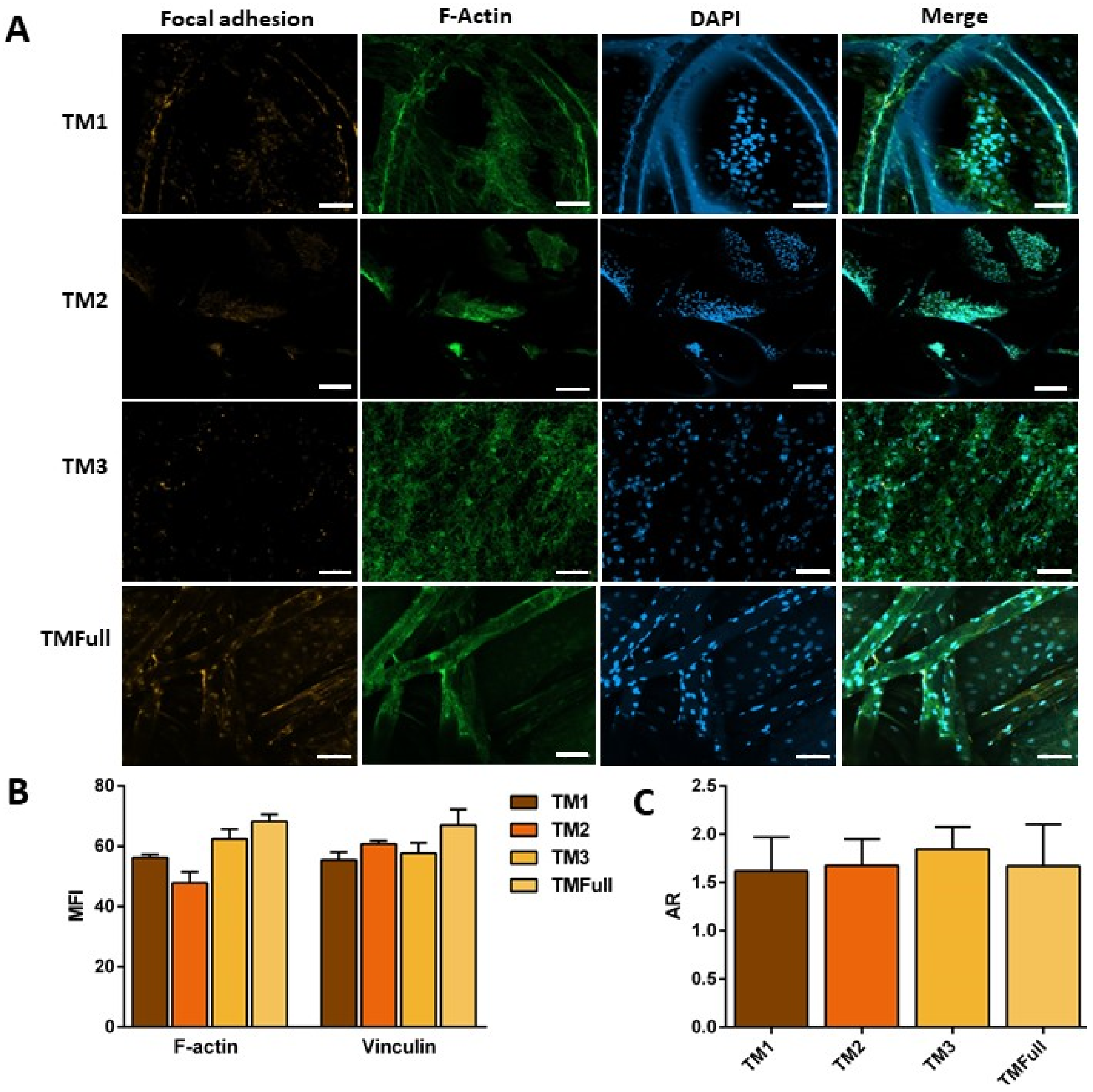

Figure S1: (A) Confocal images of HTM cells after 14 days of culture on different scaffolds: TM1, TM2, TM3, and TMFull. The first channel represents the focal adhesions (vinculin first antibody and cy3 conjugate), the second channer represents phalloidin F-actin fiber staining (AlexaFluor 488), third column DAPI for nuclei staining and the last one the merge. Scale bars: 100 μm. (B) Quantification of confocal images and mean fluorescence intensity (MFI) and (C) nucleus aspect ratio.

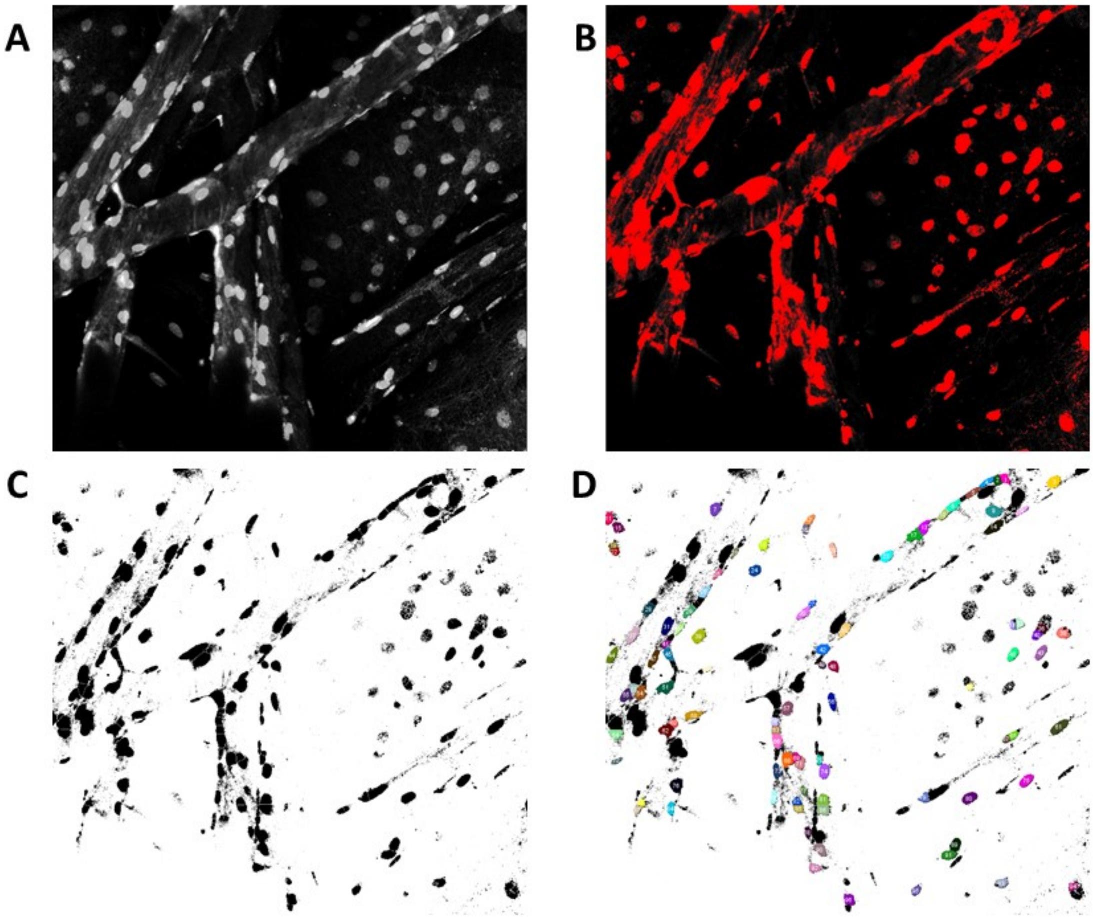

Figure S2: exemplary image processing used for nuclei aspect ratio quantification. (A) Original nuclei image stained with DAPI. (B) Image made binary. (C) Image after applying "watershed" function. (D) Nuclei considered in the calculation after applying size exclusion limit (50-150 µm). Note that the nuclei in case of clamps of cells or overlapping were excluded from the calculation.
